# Reciprocal Effects of Antiretroviral Drugs Used To Treat HIV Infection on the Fibroblast Growth Factor 21/β-Klotho System

**DOI:** 10.1128/AAC.00029-18

**Published:** 2018-05-25

**Authors:** Ricardo Moure, Pere Domingo, Joan Villarroya, Laura Gasa, José M. Gallego-Escuredo, Tania Quesada-López, Samantha Morón-Ros, Alberto F. Maroto, Gracia M. Mateo, Joan C. Domingo, Francesc Villarroya, Marta Giralt

**Affiliations:** a Department of Biochemistry and Molecular Biomedicine, Institut de Biomedicina (IBUB), University of Barcelona, Barcelona, Catalonia, Spain; b CIBER Fisiopatología de la Obesidad y Nutrición, Madrid, Spain; c Infectious Diseases Unit, Hospital de la Santa Creu i Sant Pau, Autonomous University of Barcelona, Barcelona, Catalonia, Spain; d Institut de Recerca Biomèdica (IRB) de Lleida, Lleida, Catalonia, Spain; e Department of Infectious Diseases, Hospital Universitari Arnau de Vilanova, Lleida, Catalonia, Spain; f Department of Infectious Diseases, Hospital Universitari de Santa María, Lleida, Catalonia, Spain; g Universitat de Lleida, Lleida, Catalonia, Spain

**Keywords:** antiretroviral drug, FGF21, β-Klotho, ER stress, hepatocyte, adipocyte

## Abstract

Following antiretroviral therapy, HIV-infected patients show increased circulating levels of the antidiabetic hormone fibroblast growth factor 21 (FGF21). In contrast, the expression of the FGF21-obligatory coreceptor β-Klotho (KLB) is reduced in target tissues. This situation is comparable to the FGF21 resistance status observed in obesity and type 2 diabetes. Here, we performed the first systematic study of the effects of distinct members of different antiretroviral drug classes on the FGF21/KLB system in human hepatic, adipose, and skeletal muscle cells. Most protease inhibitors and the nonnucleoside reverse transcriptase inhibitor efavirenz induced FGF21 gene expression. Neither nucleoside reverse transcriptase inhibitors nor the viral entry inhibitor maraviroc had any effect. Among the integrase inhibitors, elvitegravir significantly induced FGF21 expression, whereas raltegravir had minor effects only in adipose cells. In human hepatocytes and adipocytes, known target cells of FGF21 action, efavirenz, elvitegravir, and the lopinavir-ritonavir combination exerted inhibitory effects on KLB gene expression. Drug treatments that elicited FGF21 induction/KLB repression were those found to induce endoplasmic reticulum (ER) stress and oxidative stress. Notably, the pharmacological agents thapsigargin and tunicamycin, which induce these stress pathways, mimicked the effects of drug treatments. Moreover, pharmacological inhibitors of either ER or oxidative stress significantly impaired lopinavir–ritonavir-induced regulation of FGF21, but not KLB. In conclusion, the present *in vitro* screen study identifies the antiretroviral drugs that affect FGF21/KLB expression in human cells. The present results could have important implications for the management of comorbidities resulting from side effects of specific antiretroviral drugs for the treatment of HIV-infected patients.

## INTRODUCTION

Successful treatment of HIV-infected patients with antiretroviral therapies (ART) has largely transformed HIV infection to a chronic medical condition (1). ART regimens can achieve strong virological control, but long-term exposure to ART, as often occurs in the growing population of older HIV patients, results in increased metabolic complications (2). Insulin resistance, metabolic syndrome, enhanced cardiovascular risk, and even overt lipodystrophy are common alterations in ART-treated HIV-infected patients (3). Several classes of antiretroviral drugs appear especially likely to elicit these alterations. For example, nucleoside analogs such as zidovudine and stavudine, which inhibit reverse transcriptase, appear to particularly exacerbate lipodystrophy, whereas protease inhibitors (PIs) have been implicated in altered glucose homeostasis (4). The molecular mechanisms that account for these toxicities are incompletely understood. More recently developed drugs (i.e., integrase inhibitors, viral entry inhibitors, and novel reverse transcriptase inhibitors and PIs) appear less prone to cause overt lipodystrophy. However, their impact on metabolic homeostasis in patients is still unclear (5).

Several studies on distinct cohorts of HIV patients have reported alterations in the fibroblast growth factor 21 (FGF21) endocrine system that manifest as abnormally high levels of FGF21 (6–8). FGF21, a hormone that was recently found to play a key role in glucose and lipid homeostasis, acts as an antidiabetic and possibly antiobesity factor (9). Several recent pilot studies have reported improvement in lipidemia and body weight after short-term treatment of obese/diabetic volunteers with FGF21 analogs (10, 11). Long-acting FGF21 analogs and agonists that mimic FGF21 action are under investigation in clinical trials in patients with obesity and diabetes (ClinicalTrials registration no. NCT02413372, NCT02538874, NCT2593331, NCT02708576, and NCT03060538). FGF21 is mainly produced in the liver and targets adipose tissue (and possibly that of the liver as well), promoting glucose uptake and oxidation (12). The effects of FGF21 are mediated by FGF receptors (FGFRs) that must interact with the cell surface protein β-Klotho (KLB) to form an FGF21-responsive receptor complex. Thus, the KLB coreceptor is essential for FGF21 action on target tissues (13). The paradoxically high FGF21 levels in HIV patients are associated with a downregulation of the molecular mediator of cellular FGF21 action, KLB. This scenario, which is reminiscent of “FGF21 resistance,” is analogous to the situation found in obesity and type 2 diabetes (14, 15). It has been suggested that alterations in the FGF21 endocrine system involving liver and adipose tissues could be a major mechanism responsible for eliciting metabolic alterations in HIV patients (8, 13, 16). In this context, it has recently been reported that high FGF21 levels in HIV patients are significantly associated with altered bone homeostasis (17), consistent with previous indications of potential deleterious effects of high FGF21 levels on bone in experimental rodent models (18). Moreover, skeletal muscle, a tissue that does not express significant amounts of FGF21 in healthy individuals, shows increased expression of FGF21 under conditions in which muscle experiences mitochondrial oxidative stress (16, 19, 20), and a recent study reported enhanced expression of FGF21 in muscle from HIV patients in association with metabolic alterations (7).

Previous studies on distinct HIV patient cohorts have failed to show significant associations between abnormally high FGF21 levels and specific drugs included in ART cocktails, possibly owing to the limited number of patients and diversity of ART regimens prior to and during the study. However, several intracellular processes, including endoplasmic reticulum (ER) stress and oxidative stress, are known to induce hepatic expression of FGF21 (21–23), and there are reports that several antiretroviral drugs or drug classes (e.g., efavirenz and PIs) promote such processes (24–26). To date, less is known about molecular agents causing disturbances in the response to FGF21 in the target tissues. It has been reported that proinflammatory signaling in adipose tissue negatively regulates KLB expression (27). Then, inflammation in adipose tissue, a common condition in obesity, diabetes, and HIV lipodystrophy, may contribute to impaired FGF21 responsiveness in adipocytes.

Studies on the effects of antiretroviral drugs on human cells in culture have proven to be useful for the initial *in vitro* assessment of the potential of drugs to disturb metabolism (28–36). These studies have reported effects of several antiretroviral drugs on adipogenesis, senescence, mitochondrial toxicity, and ER stress, but none investigated their actions on the FGF21 system. In the present study, we hypothesized that antiretroviral drugs could alter the FGF21/KLB system by affecting their expression in human hepatic, adipose, and skeletal muscle cells. If so, this *in vitro* approach could be used to screen currently used antiretroviral drugs for their potential risk to cause FGF21/KLB toxicity. Here, we report a systematic analysis of the capacity of antiretroviral drugs, both “classical” and recently developed, to cause alterations in the FGF21/KLB system. That is, we analyzed their potential to promote FGF21 expression and KLB downregulation—the two key events associated with a disturbed FGF21 system in patients—in human hepatic, adipose, and muscle cells. The effects of drug-induced ER stress and oxidative stress on these alterations were also explored.

## RESULTS

### Effects of antiretroviral drugs on FGF21 and KLB expression in human hepatic cells.

**FIG 1:**
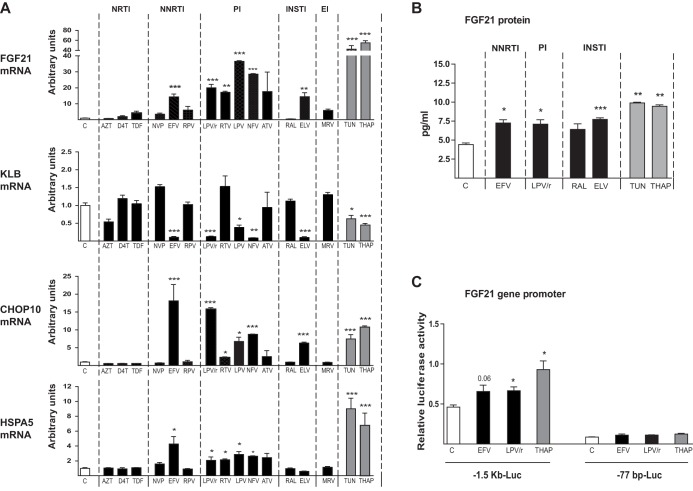
Effects of antiretroviral drugs on the expression of *FGF21*, KLB, *CHOP10*, and *HSPA5* mRNAs and on *FGF21* promoter activity in human hepatic HepG2 cells. Cells were exposed, when indicated, to the following drugs: zidovudine (AZT), 100 μM; stavudine (D4T), 100 μM; tenofovir disoproxil fumarate (TDF), 5 μM; nevirapine (NVP), 20 μM; efavirenz (EFV), 50 μM; rilpivirine (RPV), 10 μM; lopinavir-ritonavir 4:1 (LPV/r), 20 μM; ritonavir (RTV), 20 μM; lopinavir (LPV), 20 μM; nelfinavir (NFV), 20 μM; atazanavir (ATV), 50 μM; raltegravir (RAL), 50 μM; elvitegravir (ELV), 50 μM; maraviroc (MRV), 4 μM; tunicamycin (TUN), 2 μM; thapsigargin (THAP), 2 μM. (A) mRNA levels are presented as means ± SEM from 4 to 5 independent experiments and are expressed relative to values for control cells (defined as 1). (B) FGF21 protein levels in cell culture medium. (C) Luciferase activity in HepG2 cells transiently transfected with plasmid constructs in which luciferase is driven by the −1,497/+5 (−1.5 kb-Luc) or −77/+5 (−77 bp-Luc) 5′ regions of the *FGF21* gene. Cells were treated for 24 h with the indicated concentrations of drugs: EFV, 50 μM; LPV/r, 20 μM; THAP, 2 μM. Data are normalized to Renilla luciferase activity driven by the cotransfected pRL-CMV plasmid. Data are means ± SEM from 4 to 5 independent experiments. *, *P* < 0.05; **, *P* < 0.01, and ***, *P* < 0.001 for each drug treatment versus control.

To determine the possible effects of antiretroviral drugs on the FGF21 system, we first analyzed human hepatocytes, the main cellular source of FGF21 as well as a potential cellular target of FGF21 action. Among the antiretroviral drugs tested in HepG2 hepatic cells, all PIs, including the lopinavir-ritonavir 4:1 combination, elicited a robust induction of FGF21 expression (Fig. 1A). Neither the classical nucleoside reverse transcriptase inhibitors (NRTIs) zidovudine and stavudine nor the nucleotide analog tenofovir altered *FGF21* mRNA expression. Among nonnucleoside reverse transcriptase inhibitors (NNRTIs), efavirenz markedly induced *FGF21* expression, whereas neither nevirapine nor rilpivirine showed significant effects. Among integrase inhibitors (INSTIs), elvitegravir significantly induced *FGF21* expression, whereas raltegravir had no effect. The viral entry inhibitor maraviroc also had no effect. A parallel assessment of the effects of these drugs on *KLB* expression revealed a reciprocal pattern of alterations: most antiretrovirals that induced *FGF21* (i.e., most PIs, efavirenz, and elvitegravir) repressed *KLB* expression. *KLB* expression was unaffected by drugs that did not alter *FGF21* expression (Fig. 1A).

The induction of *FGF21* and repression of *KLB* observed in response to PIs and efavirenz, which are known to induce ER stress and oxidative stress in several cell types, including hepatocytes (24, 26), prompted us to examine whether these drugs also altered the expression of the genes encoding C/EBP-homologous protein 10 (*CHOP10*) and heat shock protein 5 (*HSPA5*), markers of ER stress and/or oxidative stress (37, 38). These analyses showed that the effects of drugs on *CHOP10* and *HSPA5* expression markedly paralleled their effects on *FGF21* expression, as evidenced by the significant upregulation of both *CHOP10* and *HSPA5* mRNAs in response to PIs, efavirenz, and elvitegravir (Fig. 1A). In addition, we treated HepG2 cells with tunicamycin and thapsigargin, two known inducers of ER stress. Both agents strongly induced *CHOP10* and *HSPA5* expression and elicited robust reciprocal effects on *FGF21* expression (induction) and *KLB* expression (repression)—the same effects produced by the aforementioned antiretroviral drugs (Fig. 1A).

The main changes found for *FGF21* mRNA expression were reflected in FGF21 protein levels released by cells to culture. Thus, efavirenz, the lopinavir-ritonavir combination, and elvitegravir caused a significant increase in FGF21 levels in HepG2 culture medium, whereas raltegravir had no effect (Fig. 1B). The ER stress/oxidative stress inducers tunicamycin and thapsigargin caused the highest induction in FGF21 protein release by HepG2 cells (Fig. 1B).

After this initial screening, we evaluated the concentration dependence of the effects of the drugs that had been found to alter the FGF21 system at a single relatively high dose in the study above (see Fig. S1 in the supplemental material); raltegravir was used as an ineffective drug control. Efavirenz, elvitegravir, and the lopinavir-ritonavir combination caused reciprocal concentration-response effects, inducing *FGF21* and suppressing *KLB*. With one exception, we also found that these drugs induced a concentration-dependent induction of *CHOP10* and *HSPA5* expression, similar to that found for *FGF21* induction and *KLB* repression. The exception was efavirenz, which induced expression of ER stress/oxidative stress markers only at the highest concentration tested (50 μM), despite the fact that lower concentrations (20 μM) were sufficient to alter *FGF21* and *KLB* expression. Raltegravir did not alter *FGF21* or *KLB* mRNA expression, or *CHOP10* or *HSPA5* mRNA expression at any concentration tested.

To further assess FGF21 induction, we monitored the transcriptional activity of the *FGF21* promoter after transfecting HepG2 cells with an *FGF21* promoter-luciferase reporter construct in which luciferase activity is driven by the 5′ region (1.5 kb) of the *FGF21* gene (Fig. 1C). These analyses showed that thapsigargin, the positive control for ER stress induction, and the drugs efavirenz and lopinavir-ritonavir significantly induced *FGF21* promoter activity; this effect was lost when transfections were performed using a construct comprising the −77 bp site in which most of the 5′ noncoding region of the *FGF21* gene was deleted. These results demonstrate that ER stress-inducing drugs and antiretroviral drugs that elicit increased *FGF21* mRNA expression act through the induction of *FGF21* gene transcription.

### Effects of antiretroviral drugs on FGF21 and KLB expression in human adipocytes.

**FIG 2:**
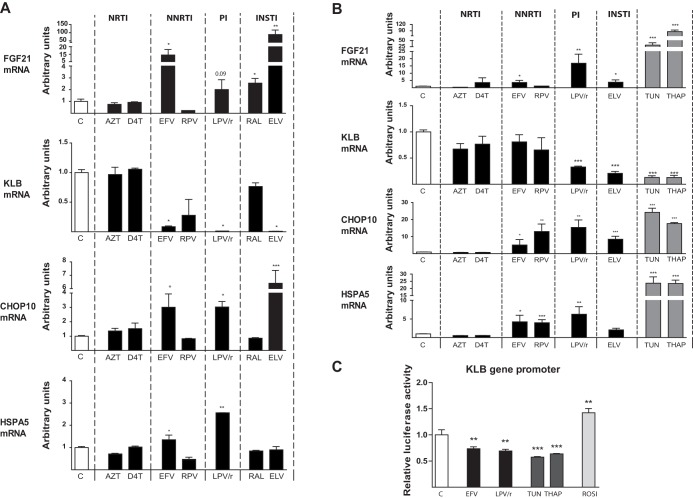
Effects of antiretroviral drugs on the expression of *FGF21*, *KLB*, *CHOP10*, and *HSPA5* mRNAs in human SGBS adipocytes and on *KLB* promoter activity in adipogenic cells. (A) SGBS human preadipocytes were differentiated in culture into adipocytes in the presence of the following drugs: zidovudine (AZT), 50 μM; stavudine (D4T), 50 μM; efavirenz (EFV), 5 μM; rilpivirine (RPV), 10 μM; lopinavir-ritonavir 4:1 (LPV/r), 20 μM; raltegravir (RAL), 5 μM; elvitegravir (ELV), 5 μM. (B) SGBS human adipocytes were differentiated in culture into adipocytes and treated for 24 h with the following drugs: AZT, 100 μM; D4T, 100 μM; EFV, 20 μM; RPV, 15 μM; LPV/r, 20 μM; ELV, 20 μM; tunicamycin (TUN), 2 μM; thapsigargin (THAP), 2 μM. Data are presented as means ± SEM from 4 to 5 independent experiments and are expressed relative to values for control cells (defined as 1). (C) Luciferase activity in adipogenic HIB-1B cells transiently transfected with a plasmid construct in which luciferase is driven by the −1,055/+45 region of the *KLB* gene. Cells were treated with the following drugs: EFV, 50 μM; LPV/r, 20 μM; TUN, 2 μM; THAP, 2 μM; rosiglitazone (ROSI), 2 μM. Data are normalized to Renilla luciferase activity driven by the cotransfected pRL-CMV plasmid. *, *P* < 0.05; **, *P* < 0.01, and ***, *P* < 0.001 for each drug treatment versus control.

Next, we analyzed the effects of antiretroviral drugs on adipocytes, the main cellular target of FGF21. Human Simpson-Golabi-Behmel Syndrome (SGBS) adipocytes undergoing adipogenic differentiation were exposed to the panel of antiretroviral drugs belonging to distinct classes. As was the case for hepatic cells, for this initial screen, the drugs were used at concentrations known to be nontoxic in this cell type, and *FGF21* and *KLB* mRNA expression levels were determined. Under control conditions, SGBS adipocytes expressed very low levels of *FGF21* mRNA, in agreement with the almost undetectable expression of FGF21 in human adipose tissue (13, 39, 40). However, we found that the NNRTI efavirenz and the INSTIs elvitegravir and raltegravir significantly induced *FGF21* mRNA expression (Fig. 2A); treatment with lopinavir-ritonavir also produced a trend towards increased *FGF21* expression, although the differences did not reach statistical significance (*P* = 0.09). Again, these drugs exerted reciprocal repressive effects on *KLB* mRNA expression. As was the case for hepatic cells, they also induced effects on the expression of the ER stress/oxidative stress markers *CHOP10* and *HSPA5* that largely paralleled their effects on *FGF21* expression; the one exception was elvitegravir, which had no effect on *HSPA5* expression (Fig. 2A). A concentration-response analysis of the drugs that showed significant effects at single concentrations indicated that efavirenz caused overt reciprocal *FGF21* induction/*KLB* repression effects at 5 μM, lopinavir-ritonavir at 20 μM, and elvitegravir in the 1 to 2 μM range (see Fig. S2). The concentration-response effects of the three types of drug treatments remarkably paralleled those for the induction of *CHOP10*. However, only lopinavir-ritonavir induced the ER stress marker *HSPA5*.

Considering that the effects of treating adipocytes with drugs throughout their adipogenic differentiation may cause secondary effects related to alterations in the overall differentiation process, we used a second experimental paradigm in which fully differentiated human adipocytes were exposed to the drugs for 24 h. The results of these experiments were very similar to those found previously in adipogenic cells chronically treated with drugs: very low basal expression of *FGF21* and significant *FGF21* induction and *KLB* repression in response to lopinavir-ritonavir and elvitegravir (Fig. 2B). We also found that the same drugs that induced *FGF21* expression and *KLB* repression induced the ER stress/oxidative stress markers *CHOP10* and *HSPA5*; the one exception was the absence of an effect of elvitegravir on *HSPA5*. The ER stress inducers thapsigargin and tunicamycin mimicked the *FGF21* induction and *KLB* repression elicited by these drugs. Notably, in this experimental setting, rilpivirine caused a significant induction of *CHOP10* and *HSPA5* expression, but this did not translate into changes in *FGF21* or *KLB* gene expression (Fig. 2B). As was the case in the first experimental setting, a concentration-response analysis showed that efavirenz, lopinavir-ritonavir, and elvitegravir caused minor effects on *FGF21* expression that paralleled changes in *CHOP10* and *HSPA5* expression, but only at high concentrations (10 to 20 μM) (see Fig. S3).

The low range of *FGF21* mRNA expression in human adipocytes was reflected in the very low levels of FGF21 in the cell culture medium, which precluded reliable measurements of FGF21 concentration changes in response to drugs, in contrast with that in hepatic and muscle cells.

Considering that *KLB* gene expression is particularly sensitive to the differentiation status of adipocytes, we investigated whether the repression of *KLB* mRNA expression induced by drugs was attributable to impaired *KLB* gene transcription. To this end, we transfected preadipocytes with a luciferase reporter plasmid driven by the *KLB* gene promoter and exposed the cells to drugs. This analysis showed that whereas rosiglitazone (used as a positive control for *KLB* gene regulation) (27, 41) induced *KLB* gene transcription, lopinavir-ritonavir and efavirenz significantly downregulated *KLB* promoter activity, as was also the case for the ER stress positive controls, tunicamycin and thapsigargin (Fig. 2C).

### Effects of antiretroviral drugs on FGF21 expression in human skeletal muscle cells.

**FIG 3:**
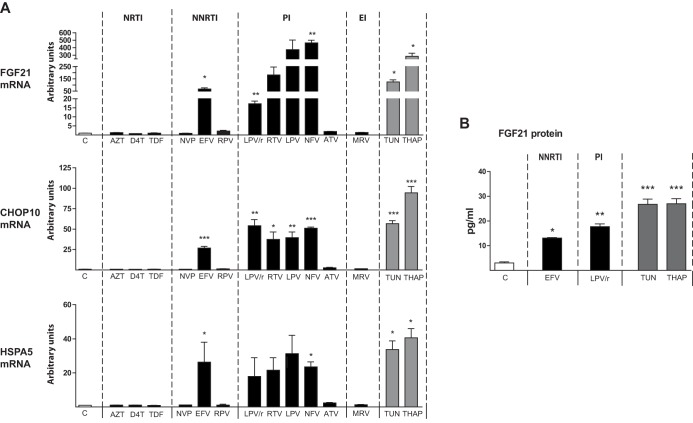
Effects of antiretroviral drugs, tunicamycin, and thapsigargin on the expression of *FGF21*, *CHOP10*, and *HSPA5* mRNAs and FGF21 release into the cell culture medium in human LHCN-M2 skeletal muscle cells differentiated in culture. LHCN-M2 myotubes differentiated in culture were treated for 24 h, as indicated, with the following drugs: zidovudine (AZT), 100 μM; stavudine (D4T), 100 μM; tenofovir disoproxil fumarate (TDF), 5 μM; nevirapine (NVP), 20 μM; efavirenz (EFV), 50 μM; rilpivirine (RPV), 10 μM; lopinavir-ritonavir 4:1 (LPV/r), 20 μM; ritonavir (RTV), 20 μM; lopinavir (LPV), 20 μM; nelfinavir (NFV), 20 μM; atazanavir (ATV), 50 μM; maraviroc (MRV), 4 μM; tunicamycin (TUN), 2 μM; thapsigargin (THAP), 2 μM. (A) mRNA levels are presented as means ± SEM from 4 to 5 independent experiments and are expressed relative to values for control cells (defined as 1). (B) FGF21 protein levels in cell culture medium. Data are presented as means ± SEM from 4 to 5 independent experiments. *, *P* < 0.05; **, *P* < 0.01, and ***, *P* < 0.001 for each drug treatment versus control.

Although skeletal muscle is not considered to be a source or target of FGF21, a recent report indicated that FGF21 expression is enhanced in muscle from HIV patients (7), a phenomenon also reported in other conditions of skeletal muscle stress such as mitochondrial diseases (19, 20). *KLB* expression was virtually undetectable in differentiated human LHCN-M2 skeletal muscle cells, as expected. *FGF21* expression was also very low under control conditions, but PIs (with the exception of atazanavir) and efavirenz caused a dramatic induction of *FGF21* expression, whereas NRTIs, NNRTIs (other than efavirenz), and maraviroc had no effect (Fig. 3A). Efavirenz and the same PIs that caused *FGF21* upregulation induced the expression of *CHOP10* and *HSPA5*. Thapsigargin and tunicamycin also induced *FGF21* expression in muscle cells.

Efavirenz, the lopinavir-ritonavir combination, and the ER stress/oxidative stress inducers thapsigargin and tunicamycin caused a significant induction of the release of FGF21 protein by LHCN-M2 myotubes into culture medium, paralleling the results found for *FGF21* mRNA expression (Fig. 3B).

### Effects of inhibitors of ER stress and oxidative stress on the action of antiretroviral drugs on FGF21 and KLB expression in human adipocytes.

**FIG 4:**
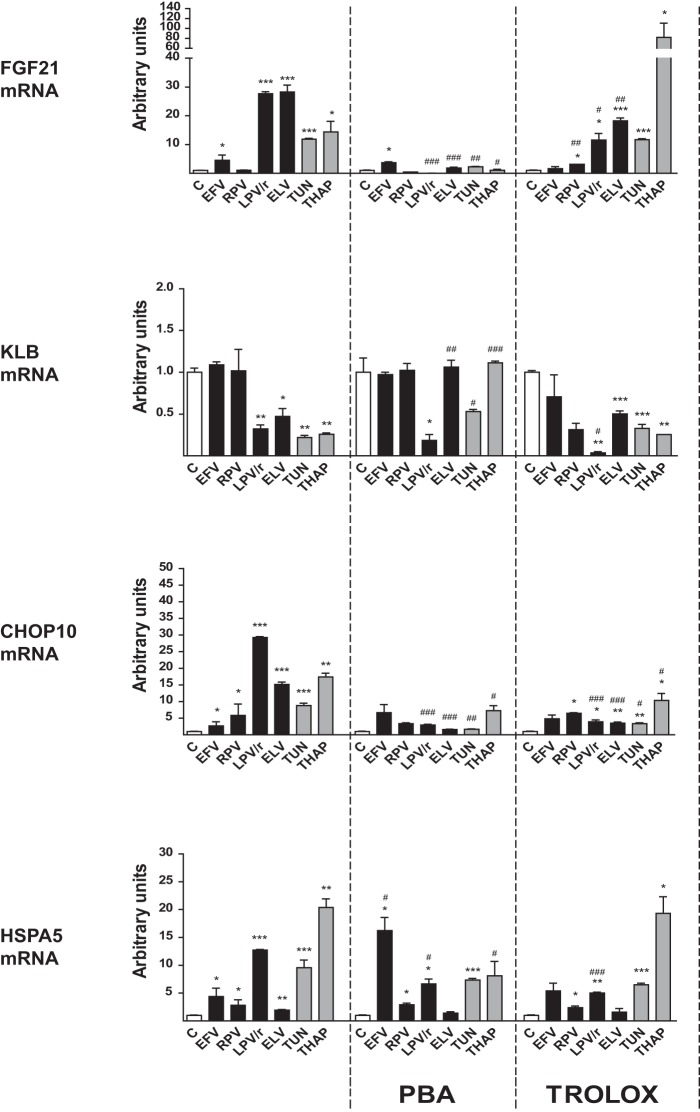
Effects of the ER stress-response inhibitor PBA and the antioxidant Trolox on the actions of antiretroviral drugs on the expression of *FGF21*, *KLB*, *CHOP10*, and *HSPA5* mRNAs in human SGBS adipocytes differentiated in culture. SGBS human preadipocytes were differentiated in culture into adipocytes and treated for 24 h with the following drugs: efavirenz (EFV), 20 μM; rilpivirine (RPV), 10 μM; lopinavir-ritonavir 4:1 (LPV/r), 20 μM; elvitegravir (ELV) 10 μM; tunicamycin (TUN), 2 μM; thapsigargin (THAP), 2 μM. Where indicated, cells were cultured in the presence of 2 mM PBA or 1 mM Trolox. Data are presented as means ± SEM from 4 to 5 independent experiments and are expressed relative to values for control cells (defined as 1). *, *P* < 0.05; **, *P* < 0.01, and ***, *P* < 0.001 for each drug treatment versus control; #, *P* < 0.05; ##, *P* < 0.01; ###, *P* < 0.001 for the effects of PBA or Trolox.

Our data demonstrating that several antiretroviral drugs, mainly PIs (e.g., lopinavir-ritonavir) and efavirenz, acted in the same manner as ER stress/oxidative stress to cause the induction of FGF21 expression and repression of KLB expression strongly suggest that ER stress and oxidative stress drive the reciprocal alterations in the FGF21 system. To test this, we used 4-phenylbutyrate (PBA), an inhibitor of ER stress, and 6-hydroxy-2,5,7,8-tetramethylchroman-2-carboxylic acid (Trolox), a vitamin E analog with antioxidant properties (Fig. 4). PBA significantly impaired the induction of ER stress. Trolox reduced thapsigargin- and tunicamycin-induced expression of *CHOP10*, but not the thapsigargin- and tunicamycin-induced expression of *HSPA5*, thus excluding HSPA5 as an oxidative stress marker; this contrasts with the dual responsiveness of CHOP10 to both ER and oxidative stress. PBA suppressed the induction of *FGF21* and repression of *KLB* expression by thapsigargin and tunicamycin, thus confirming that ER stress is capable of causing FGF21 upregulation and KLB downregulation. Both PBA and Trolox impaired the induction of *CHOP10* and *HSPA5* by lopinavir-ritonavir, suggesting that PIs are capable of causing both ER and oxidative stress. Similarly, PBA and Trolox blunted the induction of *FGF21* by lopinavir-ritonavir and elvitegravir. However, neither PBA nor Trolox affected the repression of *KLB* expression by these drugs, indicating that pathways other than ER stress/oxidative stress may be specifically involved in downregulating KLB in response to PIs. The downregulation of *KLB* elicited by elvitegravir was suppressed by PBA but was unaffected by Trolox. Efavirenz treatment had relatively modest effects in adipocytes under these conditions. Although it did significantly induce *CHOP10* and *HSPA5* expression, it caused only a minor induction of *FGF21*. These effects were relatively insensitive to PBA and Trolox, which at best exerted modest inhibitory actions.

Collectively, these data indicate that PIs, as well as elvitegravir and efavirenz, induce FGF21 expression through mechanisms involving ER and oxidative stress. However, although ER stress may induce KLB repression, other pathways are likely also involved in the repressive effect of PIs on KLB expression.

## DISCUSSION

The results of the current study indicate that some antiretroviral drugs belonging to distinct classes alter the expression of FGF21 and the FGF21 coreceptor KLB in human cells. We found that modifications in the expression of FGF21 and KLB caused by drugs parallel those observed in HIV patients undergoing antiretroviral treatment: an induction of FGF21 levels and a repression of KLB expression (6, 7, 13). Moreover, the observed alterations are systematically reciprocal: those drugs (and drug concentrations) that induce FGF21 expression and release are largely the same as those that repress KLB expression. NRTIs and the viral entry inhibitor maraviroc were universally neutral toward the FGF21/KLB system. PIs, the NNRTI efavirenz, and the INSTI elvitegravir were the drugs most capable of eliciting FGF21 induction/KLB repression effects, which otherwise followed a pattern dictated largely by the specific drug and the cell type assayed. The induction of FGF21 and repression of KLB expression in hepatic and adipose cells reflected the effects on gene transcription. Therefore, and on the basis of the remarkable concordance between the effects found here *in vitro* for several antiretroviral drugs and the alterations in the FGF21/KLB system found in HIV patients, attention should be paid to the potential of particular drugs in the ART cocktails to disturb the FGF21 system.

Subsequently, we explored the intracellular-mediated mechanisms explaining the negative effects of antiretroviral drugs on the FGF21/KLB system. In general, the drug treatments that elicited FGF21 induction/KLB repression in human hepatic, adipose, and muscle cells were also those that induced ER stress/oxidative stress. Specific pharmacological induction of ER stress/oxidative stress mimicked the effects of the drugs on the FGF21/KLB system. Moreover, the induction of ER stress/oxidative stress marker genes closely paralleled FGF21 induction and KLB repression in concentration-response analyses of lopinavir-ritonavir and elvitegravir. However, elvitegravir induced FGF21 and repressed KLB at concentrations lower than those capable of eliciting overt ER stress in hepatic cells, suggesting that pathways other than ER stress may be additionally involved in elvitegravir effects. The results obtained using inhibitors of ER stress and oxidative stress support the involvement, but not the exclusivity, of these stress pathways in the cellular actions of antiretroviral drugs on the FGF21/KLB system.

Abnormally high FGF21 levels are considered a marker of disturbed metabolism in non-HIV-infected patients with obesity, diabetes, or congenital lipodystrophy (15, 16, 42), whereas KLB repression is associated with an impairment of glucose uptake and other health effects mediated by FGF21 (27). Studies in distinct HIV patient cohorts have consistently reported that elevated FGF21 levels are associated with indicators of insulin resistance, such as homeostatic model assessment of insulin resistance (HOMA-IR), insulinemia, and glycemia (6–8, 16). Lifestyle interventions in HIV patients that achieve metabolic improvement are also associated with a decline in FGF21 levels that correlates with indications of improved energy metabolism (8). Further studies will be needed to assess if treatment only with antiretroviral drugs identified as “neutral” in our cell-based study protects HIV patients against abnormalities in the FGF21/KLB system and concomitant metabolic disturbances.

The present *in vitro* study is obviously limited with respect to the translation of results to considerations of the impact of patient treatments on the FGF21 system. The average plasma concentration of drugs in patients under standard treatment regimens is approximately 10 μM for efavirenz and lopinavir and 5 μM for elvitegravir (43–45), indicating that the effects on human adipocytes and muscle cells *in vitro* reported here occur in a concentration range that may have *in vivo* relevance. However, in our study, efavirenz and elvitegravir were added to adipocyte and myotube cell cultures in serum-free or low-serum-containing medium—culture conditions that are required for the *in vitro* differentiation of human adipocytes and muscle cells but may result in higher availability of the drugs owing to a lack of binding to proteins. Consistent with this interpretation, the concentrations of efavirenz and elvitegravir required to cause FGF21 induction/KLB repression in hepatic cells, which require 10% fetal bovine serum (FBS), were higher, implying that the presence of high levels of drug-binding proteins in the medium reduces the actual amount of drug available to the cell. On the other hand, one study that measured the accumulation of certain antiretroviral drugs in adipose tissues showed concentrations close to 100 nmol/g tissue for efavirenz and 1 nmol/g for the PI ritonavir (46); thus, intracellular concentrations, at least for efavirenz, may be higher than the plasma concentrations.

In summary, several antiretroviral drugs used in HIV therapy and belonging to distinct drug classes have been identified for the first time as triggering disturbances in the FGF21/KLB system in human hepatic, adipose, and muscle cells in culture. These drugs cause reciprocal FGF21 induction/KLB repression alterations that commonly occur in HIV patients undergoing antiretroviral treatment. ER stress/oxidative stress mechanisms appear to be involved in these effects. Considering the potential role of disruptions in the FGF21 system in metabolic, cardiovascular, and bone-related alterations, the data presented here could inspire further research designed to improve antiretroviral treatments so as to minimize the adverse effects of antiretroviral agents in HIV-1-infected patients.

## MATERIALS AND METHODS

### Reagents.

The following chemicals/drugs were used: zidovudine (GlaxoSmithKline), stavudine (Bristol-Myers Squibb), tenofovir disoproxil fumarate (Gilead Sciences), nevirapine (Boehringer Ingelheim), efavirenz (Bristol-Myers Squibb), rilpivirine (Janssen Pharmaceuticals, Inc.), ritonavir (Abbott Laboratories), lopinavir (Abbott Laboratories), nelfinavir (Agouron), atazanavir (Bristol-Myers Squibb), raltegravir (sc-364600; Santa Cruz), elvitegravir (Gilead Sciences), maraviroc (Pfizer), tunicamycin (T7765; Sigma-Aldrich Biotechnology), thapsigargin (T9033; Sigma-Aldrich), 4-phenylbutyric acid ([PBA] P21005; Sigma-Aldrich), 6-hydroxy-2,5,7,8-tetramethylchroman-2-carboxylic acid ([Trolox] 648471; Calbiochem), and rosiglitazone (ALX-350-125; Alexis Biochemicals). The reagents for cell culture were from Sigma-Aldrich, whereas media and FBS were from Life Technologies. For initial assessments of the effects of antiretroviral drugs prior to concentration-response analyses, the drugs were used at the highest concentrations that did not cause cytotoxicity in specific cell systems. The drugs were dissolved using dimethyl sulfoxide (DMSO) as the vehicle. Controls included amounts of DMSO (≤0.1% DMSO of total cell medium volume) equal to those used in drug-treated cells.

### Cell culture.

The HepG2 cell line (ATCC HB-8065), used for studies on human hepatic cells, was cultured in Dulbecco's modified Eagle medium (DMEM) containing 10% FBS. The human SGBS preadipocyte cell line (47) was used for studies on adipocytes. Human SGBS preadipocytes were cultured and differentiated to adipocytes as previously reported (36). Briefly, SGBS preadipocytes were maintained in DMEM/F12 containing 10% FBS. After the cells had become confluent, adipogenic differentiation was initiated by first incubating the cells for 6 days in serum-free medium containing 20 nM insulin, 0.2 nM triiodothyronine (T3), and 100 nM cortisol, supplemented with 25 nM dexamethasone and 500 μM 3-isobutyl-methylxantine. The cells were subsequently switched to adipogenic differentiation medium containing insulin, T3, and cortisol only and maintained for up to 10 days, at which point, more than 90% of the cells had acquired a differentiated adipocyte morphology, as evidenced by lipid droplet accumulation. Depending on the experimental design, the cells were treated with drugs throughout the 10-day differentiation process or acutely for 24 h once cells had differentiated. For studies on human skeletal muscle cells, LHCN-M2 myoblastic cells were differentiated to myotubes as previously reported (23). Briefly, myoblastic LHCN-M2 cells were cultured in DMEM/medium 199 containing 15% FBS and supplemented with 60 μg/ml ZnSO_4_, 14 μg/ml vitamin B_12_, 55 μg/ml dexamethasone, 30 μg/ml human hepatocyte growth factor, and 10 μg/ml basic fibroblast growth factor (FGF2). When cells reached ∼80% confluence, the culture medium was replaced with fresh DMEM/medium 199 supplemented with 0.5% FBS, 1 mg/ml insulin, 10 mg/ml apo-transferrin, and 55 μg/ml dexamethasone. After culturing for 2 days, the medium was replaced with DMEM/medium 199 supplemented with 55 μg/ml dexamethasone, and the cells were further cultured for 10 days, at which point, more than 90% had acquired a differentiated phenotype on the basis of their multinucleated fused myotube morphology. LHCN-M2 myotubes were exposed to drugs for 24 h. For the analysis of FGF21 protein levels, the cell culture medium was harvested 24 h after the replacement of the cell culture medium in the presence of the drugs tested.

### RNA isolation, conventional reverse transcription-PCR, and quantitative reverse transcription-PCR.

Total RNA was extracted from cells using an affinity column-based method (Macherey-Nagel) as previously described (36). Reverse transcription was performed in a total volume of 20 μl using random hexamer primers (Applied Biosystems) and 0.5 μg total RNA. PCR was performed on an ABI/Prism 7700 sequence detector system using 25 μl of a reaction mixture containing 1 μl of cDNA, 12.5 μl of TaqMan Universal PCR master mix, 250 nM probes, and 900 nM primers from an Assays-on-Demand gene expression assay mix (TaqMan; Applied Biosystems). The following Assay-on-Demand probes from Life Technologies were used: *FGF21*, Hs00173927; *KLB*, Hs00545621; *CHOP10*, Hs99999172; *HSPA5*, Hs99999174; *HPRT*, Hs99999909; *RPLP0*, Hs99999902; and 18S rRNA, Hs99999901. Controls with no RNA, primers, or reverse transcriptase were included in each set of experiments. The relative amount of individual mRNAs was calculated using the comparative (2^−ΔΔ*CT*^) method and normalized to that of the reference control gene (*HPRT* mRNA) according to the manufacturer's instructions. Each sample was run in duplicates, and the mean value of duplicates was used in calculations. Parallel calculations performed using other reference control genes (*RPLP0* mRNA and 18S rRNA) yielded essentially the same results.

### Plasmid constructions and dual luciferase reporter assays.

For studies on the transcriptional activity of *FGF21* and *KLB* genes, HepG2 hepatic cells and HIB1B adipogenic cells (48), respectively, were grown in 24-well plates and transiently transfected at ∼50% confluence with the corresponding promoter-luciferase reporter plasmids using Lipofectamine (Invitrogen). The reporter plasmid −1497-FGF21-Luc, containing a DNA fragment corresponding to positions −1,497 to +5 of the 5′ region of the mouse *Fgf21* gene linked to the firefly luciferase gene, and a −77-FGF21-Luc mutant construct containing only the −77/+5 region of the *FGF21* gene, were reported previously (48). The reporter plasmid driven by the *KLB* gene promoter, containing a DNA fragment corresponding to positions −1,055 to +45, was obtained from SwitchGear Genomics. Cells were also cotransfected with a pRL-CMV expression vector for Renilla luciferase (Promega). Each transfection condition was assayed in triplicates. The cells were incubated for 48 h after transfection and then incubated with or without drugs, as indicated in the text, for an additional 24 h before harvesting. Luciferase activity was measured on a Glomax 96 microplate luminometer using a dual luciferase reporter assay system kit (Promega). Promoter construct-driven luciferase activity was normalized to that of Renilla luciferase, used as a control for variations in transfection efficiency.

### FGF21 protein analysis.

FGF21 protein levels in cell culture media were determine using an enzyme-linked immunosorbent assay (ELISA) specific for human FGF21 (RD191108200R; Biovendor) as described previously (48). For cultures of HepG2 cells, the medium had to be concentrated (1,000 μl to 50 μl) using centrifugal filters (UFC 50195; Amicon) to achieve measurable amounts of FGF21 in the ELISAs.

### Statistical analysis.

In studies comparing high concentrations of different drugs, statistical analyses were performed using Student's *t* tests to compare the effects of each drug with that of the control. In the case of concentration-response studies, statistical analyses were performed using a one-way analysis of variance (ANOVA) followed by a Dunnett's test. Differences with *P* values of <0.05 were considered statistically significant.

## Supplementary Material

Supplemental material
