# Supplementary material for: Reciprocal Effects of Antiretroviral Drugs Used To Treat HIV Infection on the Fibroblast Growth Factor 21/β-Klotho System
Source: Antimicrob Agents Chemother. 2018 May 25;62(6):e00029-18. doi: 10.1128/AAC.00029-18 (PMC5971578; doi:10.1128/AAC.00029-18)
Supplement: Supplemental material [file supp_62_6_e00029-18__index.html]

Supplemental material 

# Reciprocal Effects of Antiretroviral Drugs Used To Treat HIV Infection on the Fibroblast Growth Factor 21/β-Klotho System

## Supplemental material

- Supplemental file 1 -

  Supplemental Figures S1 to S3

  PDF, 325K
