# Supplementary material for: Reciprocal Effects of Antiretroviral Drugs Used To Treat HIV Infection on the Fibroblast Growth Factor 21/β-Klotho System
Source: Antimicrob Agents Chemother. 2018 May 25;62(6):e00029-18. doi: 10.1128/AAC.00029-18 (PMC5971578; doi:10.1128/AAC.00029-18)

Supplementary Figure 1. Concentration-response effects of efavirenz, lopinavir/ritonavir, elvitegravir and raltegravir on the expression of FGF21, KLB, CHOP10 and HSPA5 mRNAs in human hepatic cells. HepG2 cells were treated with the indicated concentrations of drugs for 24 hours. Data are presented as means ± SEM from 4–5 independent experiments, and are expressed relative to values for control cells (defined as 1). *P < 0.05, **P < 0.01, and ***P < 0.001 for each drug treatment vs. control.

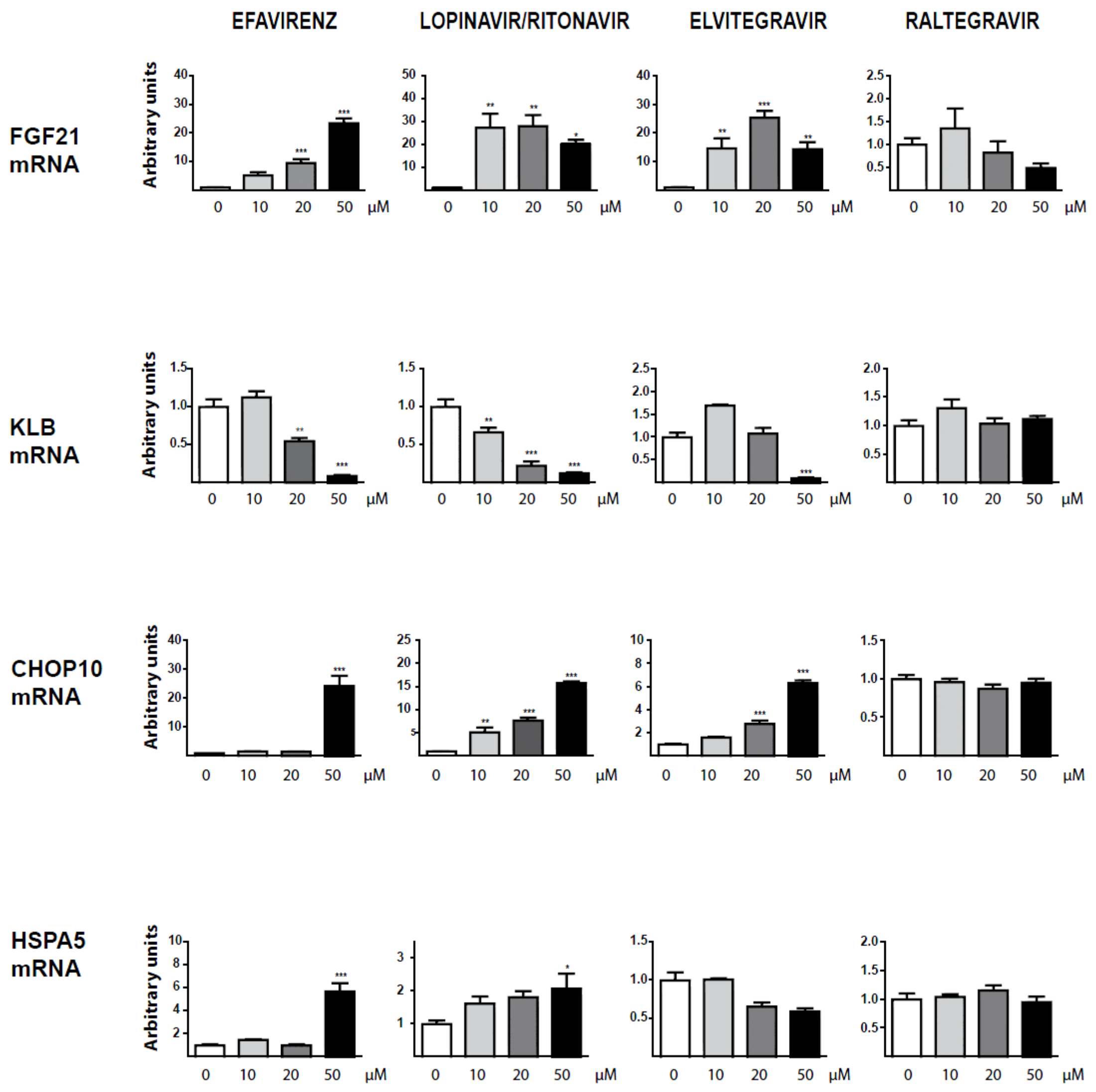

Supplementary Figure 2. Concentration-response effects of efavirenz, lopinavir/ritonavir, elvitegravir and raltegravir on the expression of FGF21, KLB, CHOP10 and HSPA5 mRNAs in human adipocytes during differentiation. SGBS human preadipocytes were differentiated in culture into adipocytes in the presence of the indicated concentrations of drugs. Data are presented as means ± SEM from 4–5 independent experiments, and are expressed relative to values for control cells (defined as 1). *P < 0.05, **P < 0.01, and ***P < 0.001 for each drug treatment vs. control.

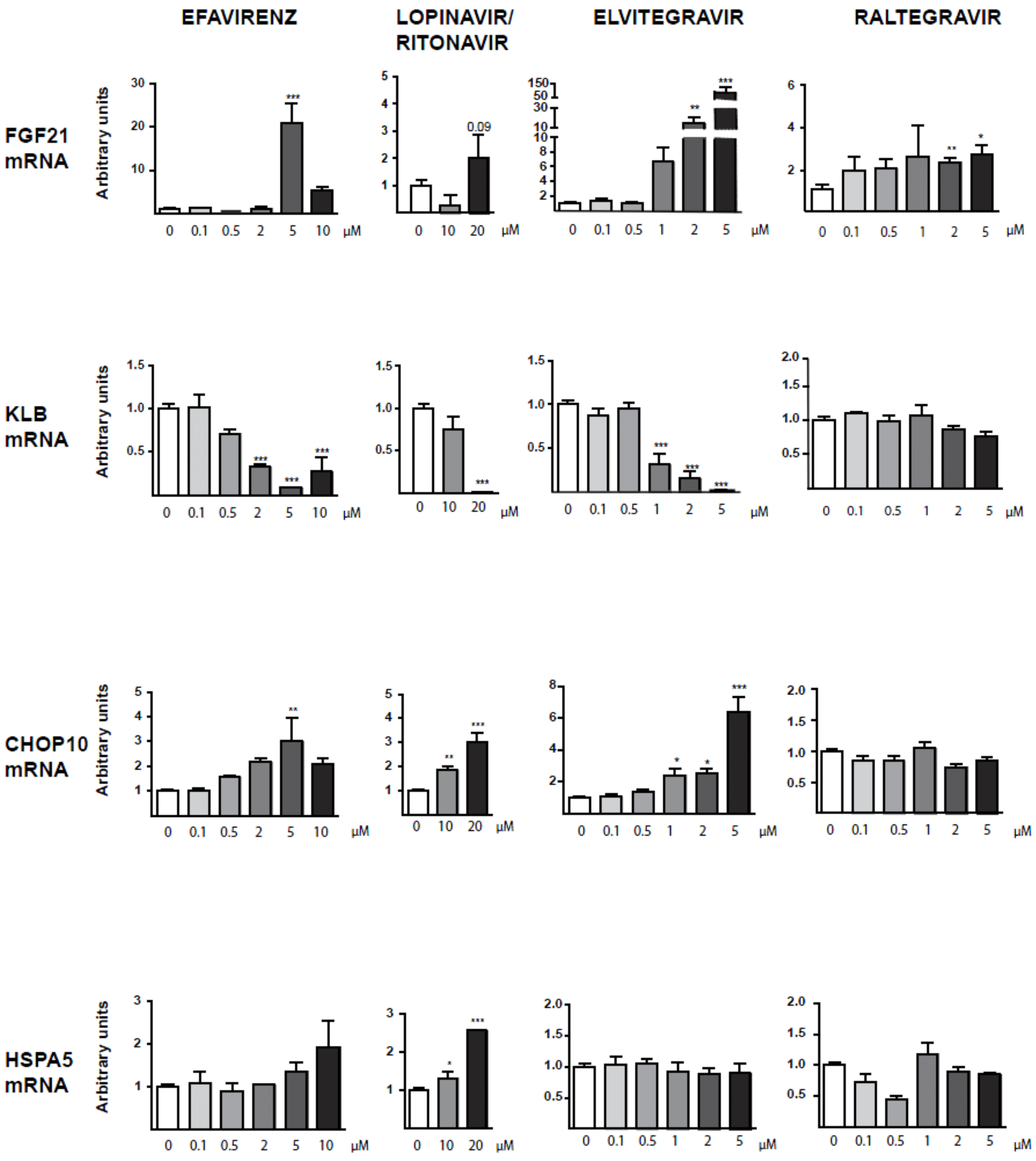

Supplementary Figure 3. Concentration-response effects of efavirenz, lopinavir/ritonavir and elvitegravir on the expression of FGF21, KLB, CHOP10 and HSPA5 mRNAs in SGBS human adipocytes differentiated in culture. SGBS human preadipocytes were differentiated in culture into adipocytes and treated with the indicated concentrations of drugs for 24 hours. Data are presented as means ± SEM from 4–5 independent experiments, and are expressed relative to values for control cells (defined as 1). *P < 0.05, **P < 0.01, and ***P < 0.001 for each drug treatment vs. control.

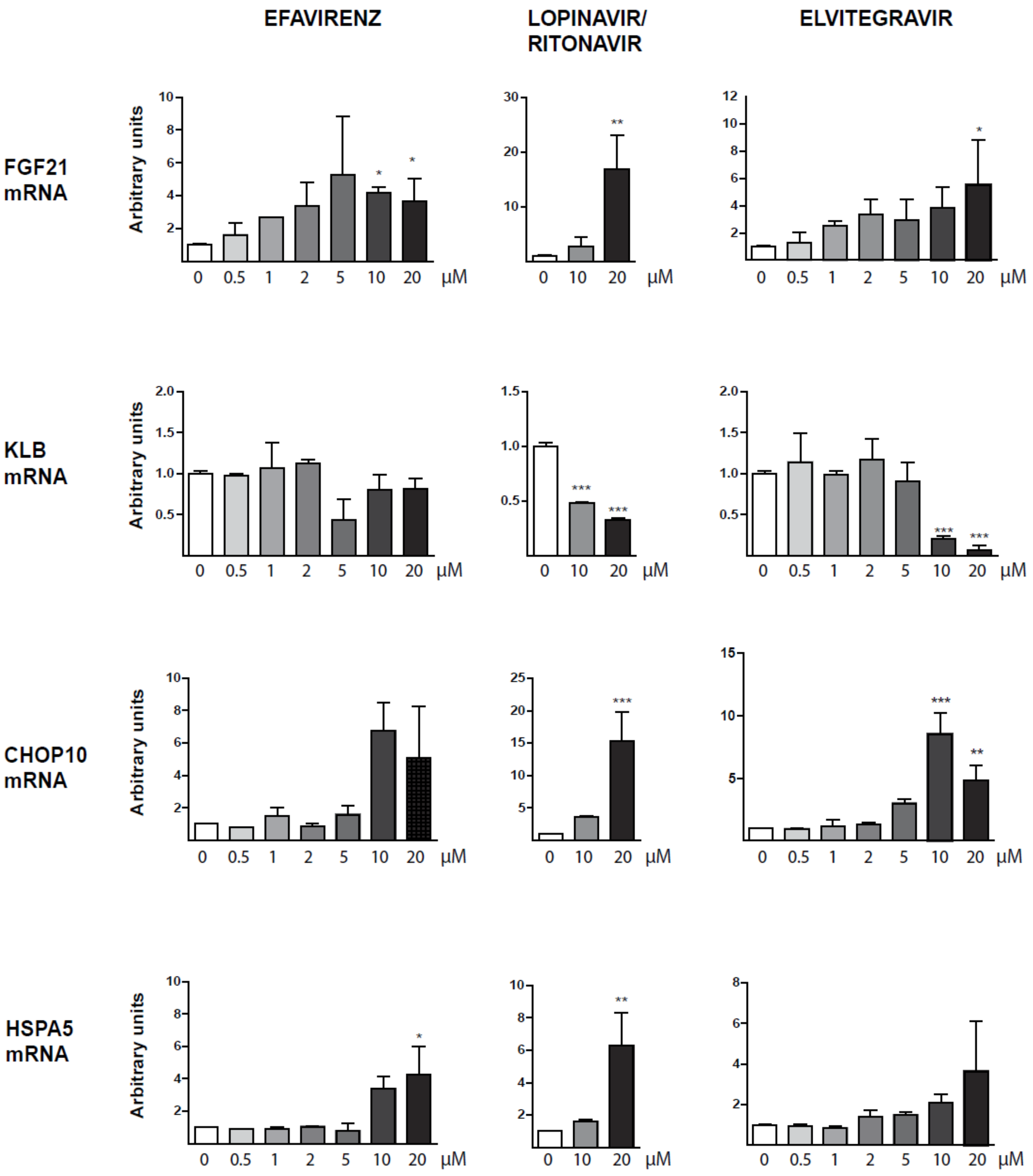

Supplement: Supplemental material [file AAC.00029-18_zac006187224s1.pdf]
